# Supplementary material for: From sequence to enzyme mechanism using multi-label machine learning
Source: BMC Bioinformatics. 2014 May 19;15:150. doi: 10.1186/1471-2105-15-150 (PMC4229970; doi:10.1186/1471-2105-15-150)
Supplement: Additional file 2 — Java code of ml2db. Additional file ml2db_code.tar.gz contains the Java source code to run the multi-label machine learning experiments and save the results to database. The code’s Javadoc is included. [file 1471-2105-15-150-S2.zip › additional file 2/ml2db/ml2db/doc/main/ml2db/DebuggingObjectOutputStream.html]

DebuggingObjectOutputStream


JavaScript is disabled on your browser.


- Overview
- Package
- Class
- Use
- Tree
- Deprecated
- Index
- Help

- Prev Class
- Next Class

- Frames
- No Frames

- All Classes

- Summary:
- Nested |
- Field |
- Constr |
- Method

- Detail:
- Field |
- Constr |
- Method


main.ml2db

## Class DebuggingObjectOutputStream

- java.lang.Object
- - java.io.OutputStream
  - - java.io.ObjectOutputStream
    - - main.ml2db.DebuggingObjectOutputStream

- All Implemented Interfaces:
  :   java.io.Closeable, java.io.DataOutput, java.io.Flushable, java.io.ObjectOutput, java.io.ObjectStreamConstants, java.lang.AutoCloseable

  ---

    

  ```
  public class DebuggingObjectOutputStream
  extends java.io.ObjectOutputStream
  ```

- - ### Nested Class Summary

    - ### Nested classes/interfaces inherited from class java.io.ObjectOutputStream

      `java.io.ObjectOutputStream.PutField`
  - ### Field Summary

    - ### Fields inherited from interface java.io.ObjectStreamConstants

      `baseWireHandle, PROTOCOL_VERSION_1, PROTOCOL_VERSION_2, SC_BLOCK_DATA, SC_ENUM, SC_EXTERNALIZABLE, SC_SERIALIZABLE, SC_WRITE_METHOD, STREAM_MAGIC, STREAM_VERSION, SUBCLASS_IMPLEMENTATION_PERMISSION, SUBSTITUTION_PERMISSION, TC_ARRAY, TC_BASE, TC_BLOCKDATA, TC_BLOCKDATALONG, TC_CLASS, TC_CLASSDESC, TC_ENDBLOCKDATA, TC_ENUM, TC_EXCEPTION, TC_LONGSTRING, TC_MAX, TC_NULL, TC_OBJECT, TC_PROXYCLASSDESC, TC_REFERENCE, TC_RESET, TC_STRING`
  - ### Constructor Summary

    Constructors

    Constructor and Description

    `DebuggingObjectOutputStream()`
  - ### Method Summary

    Methods

    | Modifier and Type | Method and Description |
    | --- | --- |
    | `void` | `writeObjectOverride(java.lang.Object obj)` |

    - ### Methods inherited from class java.io.ObjectOutputStream

      `close, defaultWriteObject, flush, putFields, reset, useProtocolVersion, write, write, write, writeBoolean, writeByte, writeBytes, writeChar, writeChars, writeDouble, writeFields, writeFloat, writeInt, writeLong, writeObject, writeShort, writeUnshared, writeUTF`
    - ### Methods inherited from class java.lang.Object

      `equals, getClass, hashCode, notify, notifyAll, toString, wait, wait, wait`

- - ### Constructor Detail


    - #### DebuggingObjectOutputStream

      ```
      public DebuggingObjectOutputStream()
                                  throws java.io.IOException
      ```

      Throws:
      :   `java.io.IOException`
  - ### Method Detail


    - #### writeObjectOverride

      ```
      public final void writeObjectOverride(java.lang.Object obj)
                                     throws java.io.IOException
      ```

      **Overrides:**
      :   `writeObjectOverride` in class `java.io.ObjectOutputStream`

      Throws:
      :   `java.io.IOException`


- Overview
- Package
- Class
- Use
- Tree
- Deprecated
- Index
- Help

- Prev Class
- Next Class

- Frames
- No Frames

- All Classes

- Summary:
- Nested |
- Field |
- Constr |
- Method

- Detail:
- Field |
- Constr |
- Method
